# N-acetylcysteine Counteracts Adipose Tissue Macrophage Infiltration and Insulin Resistance Elicited by Advanced Glycated Albumin in Healthy Rats

**DOI:** 10.3389/fphys.2017.00723

**Published:** 2017-09-22

**Authors:** Karolline S. da Silva, Paula R. Pinto, Nelly T. Fabre, Diego J. Gomes, Karina Thieme, Ligia S. Okuda, Rodrigo T. Iborra, Vanessa G. Freitas, Maria H. M. Shimizu, Walcy R. Teodoro, Suely K. N. Marie, Tom Woods, Margaret A. Brimble, Russell Pickford, Kerry-Anne Rye, Maristela Okamoto, Sergio Catanozi, Maria L. Correa-Giannela, Ubiratan F. Machado, Marisa Passarelli

**Affiliations:** ^1^Laboratorio de Lipides, LIM-10, Faculdade de Medicina, Hospital das Clinicas, Universidade de São Paulo São Paulo, Brazil; ^2^Laboratorio de Carboidratos e Radioimunoensaios, LIM-18, Faculdade de Medicina, Hospital das Clinicas, Universidade de São Paulo São Paulo, Brazil; ^3^Laboratorio de Biologia Celular e Molecular, LIM-15, Faculdade de Medicina, Hospital das Clinicas, Universidade de São Paulo São Paulo, Brazil; ^4^Laboratorio de Pesquisa Básica em Doenças Renais, LIM-12, Faculdade de Medicina, Hospital das Clinicas, Universidade de São Paulo São Paulo, Brazil; ^5^Laboratorio de Reumatologia, LIM-17, Faculdade de Medicina, Hospital das Clinicas, Universidade de São Paulo São Paulo, Brazil; ^6^School of Chemical Sciences and School of Biological Sciences, University of Auckland Auckland, New Zealand; ^7^Bioanalytical Mass Spectrometry Facility, University of New South Wales Sydney, NSW, Australia; ^8^Lipid Research Group, School of Medical Sciences, Faculty of Medicine, University of New South Wales Sydney, NSW, Australia; ^9^Laboratorio de Metabolismo e Endocrinologia; Instituto de Ciencias Biomedicas, Universidade de São Paulo São Paulo, Brazil

**Keywords:** advanced glycation, adipose tissue, advanced glycated albumin, insulin resistance, diabetes mellitus, macrophage differentiation

## Abstract

**Background:** Advanced glycation endproducts elicit inflammation. However, their role in adipocyte macrophage infiltration and in the development of insulin resistance, especially in the absence of the deleterious biochemical pathways that coexist in diabetes mellitus, remains unknown. We investigated the effect of chronic administration of advanced glycated albumin (AGE-albumin) in healthy rats, associated or not with N-acetylcysteine (NAC) treatment, on insulin sensitivity, adipose tissue transcriptome and macrophage infiltration and polarization.

**Methods:** Male Wistar rats were intraperitoneally injected with control (C) or AGE-albumin alone, or, together with NAC in the drinking water. Biochemical parameters, lipid peroxidation, gene expression and protein contents were, respectively, determined by enzymatic techniques, reactive thiobarbituric acid substances, RT-qPCR and immunohistochemistry or immunoblot. Carboxymethyllysine (CML) and pyrraline (PYR) were determined by LC/mass spectrometry (LC-MS/MS) and ELISA.

**Results:** CML and PYR were higher in AGE-albumin as compared to C. Food consumption, body weight, systolic blood pressure, plasma lipids, glucose, hepatic and renal function, adipose tissue relative weight and adipocyte number were similar among groups. In AGE-treated animals, insulin resistance, adipose macrophage infiltration and *Col12a1* mRNA were increased with no changes in M1 and M2 phenotypes as compared to C-albumin-treated rats. Total GLUT4 content was reduced by AGE-albumin as compared to C-albumin. NAC improved insulin sensitivity, reduced urine TBARS, adipose macrophage number and *Itgam* and *Mrc mRNA* and increased *Slc2a4* and *Ppara*. CD11b, CD206, *Ager, Ddost, Cd36, Nfkb1, Il6, Tnf*, *Adipoq, Retn, Arg, and Il12* expressions were similar among groups.

**Conclusions:** AGE-albumin sensitizes adipose tissue to inflammation due to macrophage infiltration and reduces GLUT4, contributing to insulin resistance in healthy rats. NAC antagonizes AGE-albumin and prevents insulin resistance. Therefore, it may be a useful tool in the prevention of AGE action on insulin resistance and long-term complications of DM.

## Introduction

Low-grade chronic inflammation plays an important role in the onset of insulin resistance, and adipose tissue acts as a primary site for the production of inflammatory mediators and adipokines (Savage et al., 2007). Macrophages are regarded as being particularly important to adipose tissue-triggered inflammation considering their polarization into (classically activated) M1 macrophages that secrete interleukin 6 (IL-6), interleukin 12 (IL-12), tumor necrosis factor alpha (TNFα) and monocyte chemoattractant protein 1 (MCP-1) rather than the M2 (alternatively activated) phenotype that exerts anti-inflammatory actions by the production of interleukin 4 (IL-4), interleukin 10 (IL-10) and interleukin 13 (IL-13) (Lumeng et al., 2007; Becker et al., 2012). Nonetheless, the exact mechanisms that render adipocyte tissue prone to an inflammatory milieu are not well understood and although obesity is a common hallmark of this process, it is not exclusive to it.

Advanced glycation end products (AGEs) originate from the non-enzymatic covalent reaction between glucose or other monosaccharides with lysine and arginine residues in proteins as well as the amino-terminal portion of other macromolecules including some phospholipids. A Schiff base is initially produced which progresses to a more stable Amadori Product. Inter and intramolecular rearrangements of these compounds favor the generation of highly reactive oxoaldehydes such as methylglyoxal (MGO), glyoxal (GO), and deoxyglucosone. They rapidly and irreversibly interact with macromolecules leading to AGEs formation, which alter the primary structure of proteins and its functionality. AGEs are prevalent in diabetes mellitus (DM) due to hyperglycemia but also in chronic kidney disease and inflammatory states where there is, respectively, a failure in detoxification of glycation intermediates by the kidneys and an elevated generation of oxoaldehydes, such as glycolaldehyde (GAD). In addition, the oxidation of polyunsaturated fatty acids, ketone bodies, and some amino acids lead to the formation of MGO and GO. Thus, AGEs can be generated independently of hyperglycemia and can be also obtained from exogenous sources such as tobacco and foods rich in fats and proteins cooked for a long time at high temperature (Cerami et al., 1997; Uribarri et al., 2010; Machado et al., 2014; Gomes et al., 2016).

Serum albumin represents the major protein modified by AGEs in circulation and transports exogenous AGEs after they reach circulation, mediating the deleterious actions of AGEs in many tissues (Neelofar et al., 2016). AGEs interact with the receptor for AGE (RAGE) favoring reactive oxygen species (ROS) generation, nuclear factor kappa B (NFKB) activation and the expression of inflammatory genes and *Ager* (RAGE) propagating the AGE/RAGE axis signaling and inflammation (Ramasamy et al., 2007). On the other hand, advanced glycation endproducts receptor 1 (AGER-1) antagonizes RAGE signaling and induces antioxidant defenses (Cai et al., 2006) counteracting the AGE-induced oxidative and inflammatory stress that are blamed for the metabolic memory and long-term complications of diabetes mellitus (DM) (Brownlee, 2001; Furusyo and Hayashi, 2013; Vlassara and Uribarri, 2014).

N-acetylcysteine (NAC) is a glutathione precursor with a potent antioxidant property that is able to reduce AGE plasma levels, lipid peroxidation and inflammation. In addition, NAC prevents the deleterious effects of AGEs in macrophage lipid homeostasis (Machado et al., 2014).

AGEs worsen insulin sensitization in cultured adipocytes, muscle cells and alter glucose secretion and disposal (Unoki et al., 2007; Wu et al., 2011). Nonetheless, there is little *in vivo* data on the causal relationship between AGEs and glucose metabolism derangements in adipose tissue, especially regarding macrophage infiltration and polarization. Therefore, we addressed the role played by chronic administration of AGE-albumin alone or together with NAC in healthy Wistar rats in the periepididymal adipose tissue transcriptome, histology, macrophage infiltration and differentiation as well as expression of genes related to glucose and lipid disposal that may contribute to insulin resistance and/or DM worsening. AGEs triggered insulin resistance in rats by sensitizing adipose tissue to inflammation that was related to increased adipose macrophage infiltration and a reduced GLUT4 protein level. NAC counteracted the deleterious effects of AGEs by diminishing lipid peroxidation and adipose macrophage number while increasing *Slc2a4* expression and insulin sensitivity.

## Materials and methods

### Advanced glycation of rat albumin

Rat fatty acid free albumin (RSA; A6414; Sigma-Aldrich, Saint Louis, Missouri, USA) was incubated with 10 mM glycolaldehyde (Sigma Chemical Co., St. Louis, MO, USA) for 4 days, at 37°C, under sterile conditions, in a water bath shaker, under N_2_ atmosphere, in the dark. Control (C) albumin was incubated with phosphate buffered saline (PBS), pH 7.4, alone. After extensive dialysis, samples were sterilized in a 0.22 μM filter and frozen at −80°C. Carboxymethyllysine (CML) in albumin samples was determined by ELISA (CircuLex, CycLex Co., Ltd, Nagano, Japan). In addition, to have a more accurate measurement, CML and pyrraline (PYR) were quantified by liquid chromatography/mass spectrometry (LC-MS/MS).

### Liquid chromatography–mass spectrometry (LC–MS/MS)

For protein digestion, C and AGE-albumin were incubated with pepsin, pronase E, aminopeptidase and prolidase, under argon gas, at 37°C, for 4 days, according to Rabbani et al. (2014). A gradient solution [0.1% formic acid and 5 mM ammonium formate in water (mobile phase A) and 0.01% formic acid in acetonitrile (mobile phase B)] at the flow rate of 500 μl/min (0.7 min of 95% B, 4 min of 99% A, 5.3 min of 95% B) was used to separate the enzymatic hydrolysates at 40°C (SeQuant ZIC®HILIC HPLC column (IDLX: 2.1 × 100 mm; Particle size: 3.5 μm and Pore Size: 200 A -Merck Millipore).

The HPLC system was directly coupled to a TSQ Quantum Access triple quadrupole mass spectrometer (Thermo Fisher Scientific); sheath gas pressure and auxiliary gas pressure were 30 and 15 (Thermo Fisher Scientific arbitrary units), respectively, and capillary temperature 245°C; collision-induced dissociation was performed in Q2 operated with argon at 2 kPa. For each analyte two most intense selected reaction-monitoring transitions were chosen. XCalibur software was used for the quantification and the calibration curves generated by analysis of unlabeled standard with a constant concentration of isotopically labeled standards were used to calculate the compound concentration in the samples (Woods et al., 2013).

### Animals

This study was approved by the Institutional Care and Research Advisory Committee (CAPPesq HC-FMUSP #002/14), and was performed according to the U.S. National Institutes of Health (NIH) Guide for the Care and Use of Laboratory Animals. Four-week old male Wistar rats (weighing 207.6 ± 13.83 g) randomized into four groups were intraperitoneally injected with: (1) control (C) albumin (20 mg/kg/day; *n* = 8); (2) C-albumin + NAC in drinking water (600 mg/L; Sigma A7250; St Louis Missouri, USA; *n* = 7); (3) AGE-albumin (20 mg/kg/day; *n* = 8) and (4) AGE-albumin + NAC (*n* = 7), for 90 consecutive days. The dose of C and AGE-albumin injected was based in a previous study by Coughlan et al. (2011). All animals were housed in a controlled environment (12 h light/dark), fed a chow diet and had access to water *ad libitum*. Food consumption and body weight were assessed weekly.

### Biochemistry and blood pressure measurement

Plasma total cholesterol (TC), triglycerides (TG), free fatty acid (FFA), alanine aminotransferase (ALT), aspartate aminotransferase (AST), urea, creatinine and glucose levels were determined by enzymatic techniques at baseline and at the end of the study. All determinations were carried out in plasma obtained from blood samples (500 μL) drawn from the tail vein into heparinized tubes, after a 12 h overnight fast. Serum insulin was determined by ELISA (Merck Millipore, Darmstadt, Germany). Proteinuria and thiobarbituric acid reactive substances (TBARS) were measured in 24 h urine samples as previously described (Shimizu et al., 2008). Systolic blood pressure (SBP) was assessed in conscious rats with a standard tail-cuff technique using an oscillometric method. Animals were mildly warmed for 30 min prior to SBP assessment. BP measurements were performed in rested animal. After three successive days of preconditioning to the measurement system, 12 readings were carried out over two consecutive days and averaged to obtain mean values.

### Insulin tolerance test (ITT)

At the end of 12th week, animals were anesthetized via i.p. injection with thiopental sodium (60 mg/kg of body mass –Cristália, São Paulo, Brazil). Tail blood samples were obtained (0 min) and at 4, 8, 12, and 16 min after an intravenous (i.v.; penis vein) injection of regular human insulin (100 UI/mL/kg of body mass; Eli Lilly and company, Indianapolis, EUA). The constant rate of the blood glucose disappearance (kITT) was determined by linear regression (neperian logarithm). The values at time 0 correspond to glucose levels at the beginning of the test in animals fed for 2 h.

### Immunohistochemistry and histology

Periepididymal fat was excised, briefly dried on filter paper, weighed and expressed as a percentage of body mass. Tissue was divided into two sections; one fraction was kept frozen for mRNA analysis and the other was washed in PBS, pH 7.4 and fixed in 10% formaldehyde for 24 h, for immunohistochemistry as described below. The tissue was washed in PBS, pH 7.4 and fixed in 10% formaldehyde for 24 h and embedded in paraffin (Merck, São Paulo, Brazil) for light microscopy. Slices (3–4 μm thick) were cut and underwent Hematoxylin–Eosin (H&E) staining for morphometric analysis of inflammatory cells (monucleated and polymorphonucleated cells). The number of adipocytes (average surface of 10 random sorted fields per animal) was determined by Image Pro-Plus software 6.0 (Media Cybernetics, Bethesda, MD, USA). The images were scanned in the 400 X magnification and the quantification was performed with a reticulum with 100 points and 50 lines. Data were normalized by the reticulum area in the histological field which is 1.250 μm^2^ and results expressed in μm^2^ (Gundersen et al., 1988).

The subpopulations of macrophages were identified by immunohistochemistry. The sections were deparaffinized and a 0.3% hydrogen peroxide solution was applied during for 4–5 min to inhibit endogenous peroxidase activity. Tissue sections were pretreated in citrate buffer solution, pH 6.0 and heated in a Pascal pressure cooker (125°C, for 1 min) to unmask the epitopes. The primary antibodies for immunohistochemical reactions were from Abcam (Cambridge, UK) and consisted of anti-F4/80 (Cat. # ab74383; 1:100), anti- CD11b (Cat. # ab64347; 1:200) and anti-CD206 (Cat. # ab64693; 1:1250). The reaction was revealed using biotin–streptavidin–peroxidase VECTASTAIN Elite ABC HRP Kit (Peroxidase, Mouse IgG; PK6102), according to manufacturer's instructions. The 3,3 diaminobenzidine (Sigma Chemical, St Louis, MO) was used as a chromogen. The sections were counterstained with Harris hematoxylin (Merck, Darmstadt, HE Germany). For negative controls, the primary antibody was replaced with PBS. Morphometric analysis was done using the Image-Pro Plus 6.0 system composed by an Olympus camera (Olympus Co, St Laurent, Quebec, Canada) coupled to an Olympus microscope (Olympus BX51), from which the images were sent to an LG monitor by means of a digitizing system (Oculus TCX, Coreco, Inc, St. Laurent, Quebec, Canada) and downloaded to a computer (Pentium 1330 Mhz). The number of inflammatory cells and F4/80-positive macrophages in periepididymal adipose tissue (along with the number of macrophages exhibiting M1 and M2 phenotypic markers) was determined by the point-counting technique (Gundersen et al., 1988) using a reticulum grid with 100 points distributed orthogonally on the acquired image in 10 random fields at 400 X magnification. The adipocyte number was calculated as the proportion of the number of points hitting the morphological parameters in fat tissue and the total grid area was expressed as a percentage.

### Real-time RT-qPCR analysis

Total RNA was extracted from periepididymal adipose tissue by Trizol reagent (Invitrogen Life Technologies, Carlsbad, CA, USA) and purified by RNeasy Mini Kit (Qiagen, USA). Total RNA (2 μg) was reverse transcribed to cDNA using High Capacity RNA-to-cDNA kit (Applied Biosystems, Foster City, CA, USA) according to the manufacturer's instructions. Real-Time quantitative PCR was performed by Taqman assays (Applied Biosystems, Foster City, CA, USA). The following TaqMan Gene Expression Assays were used: rat *Ager* (RAGE/advanced glycosylation end product-specific receptor; Rn01525753_g1), *Ddost* (AGER-1/dolichyl-diphosphooligosaccharide-protein glycosyltransferase non-catalytic subunit; Rn01518759_m1), *Cd36* (CD36 molecule; Rn00580728_m1), *Nfkb1* (nuclear factor kappa B subunit 1; Rn01399583_m1), *Il6* (interleukin 6; Rn01410330_m1), *Tnf* (tumor necrosis fator; Rn01525859_g1), *Adipoq* (adiponectin; Rn00595250_m1), *Retn* (resistin; Rn00595224_m1), *Slc2a4* (solute carrier family 2 member 4; Rn00562597_m1), *Ppara* (peroxisome proliferator activated receptor alpha; Rn00566193_m1), *Ccl2* (C-C motif chemokine ligand 2; Rn00580555_m1), *Mrc* (mannose receptor, C type 1; Rn01487342_m1), *Itgam* (integrin subunit alpha M; Rn00709342_m1), *Il12* (interleukin 12B; Rn00584538_m1), *Arg1* (arginase 1; Rn00691090_m1), *Col4a1* (collagen type IV alpha 1 chain; Rn01482927_m1), *Col4a2* (collagen type IV alpha 2 chain; Rn01482133_m1), *Col5a1I* (collagen type V alpha 1 chain; Rn00593170_m1), *Col5a3* (collagen type V alpha 3 chain; Rn01448295_m1), *Col12a1I* (collagen type XII alpha 1 chain; Rn01521220_m1), *Itgb8* (integrin subunit beta 8; Rn02106015_u1) in the StepOne Plus - Real Time PCR System (Applied Biosystems by Life Technologies, USA). The relative expression of each gene was normalized to the housekeeping gene *Hprt1* (hypoxanthine phosphoribosyltransferase 1; Rn01527840_m1) and relative quantification analysis was performed with StepOne Software 2.0 (Applied Biosystems) using the comparative cycle threshold (Ct) (2−^ΔΔCt^) method (Livak and Schmittgen, 2001).

### Adipose tissue transcriptome: RNA-sequencing data analysis

Total mRNA of periepididymal adipose tissue with adequate RNA quantity and quality (>100 ng/μL and RNA integrity number [RIN] ≥7) was converted to a library of cDNA fragments with adaptors attached (DNA basis) using *TruSeq*® *Stranded Total RNA with Ribo Zero Gold* kit (Illumina, California, USA) according to the manufacters' instructions for amplification and sequencing in HiSeq® 2500 *Sequencing System* (Illumina). FAST-QC (http://www.bioinformatics.babraham.ac.uk/projects/fastqc/) quality checks were done to determine high quality base pairs, and *bbduk* (https://sourceforge.net/projects/bbmap/) was applied to trim low quality reads and adapters (Babraham Bioinformatics, 2017). Data was aligned using the DeSeq2 Version 3.5 (http://www.bioconductor.org/packages/release/bioc/html/DESeq2.html; Love et al., 2014) to the ensemble HG38 reference genome obtained from Ensembl and quantified with feature counts (Liao et al., 2014). Quality check of aligned data was done with RNA-SEQC (DeLuca et al., 2012) and showed high quality alignment and data. Non-expressed genes were flagged with DAFS (George and Chang, 2014) and removed from downstream analysis. The signal adjusted *p*-value > 0.1, fold change < 0.04, micro-RNA and pseudogenes were excluded. Hypo or overexpressed genes were identified by WebGestalt (WEB-based Gene Set Analysis Toolkit) which covers enrichment functional categories (GO) and GSEA (Gene Set Enrichment Analysis). Then, the protein network was determined by STRING (Search Tool for the Retrieval of Interacting Genes), version 9.0, using an interaction score 0.400—medium confidence.

### Western blotting analysis

Samples of periepididymal fat pad were homogenized in sucrose buffer pH 7.4 (10 mmol/l Tris–HCl, 1 mmol/l EDTA and 250 mmol/l sucrose) and centrifuged at 1.000 × *g* for 15 min. Fat cake was discarded and the infranatant (fat-free extract, FFE) used for western blotting. Protein (30 μg) from each sample was subjected to electrophoresis [10% T sodium dodecyl sulfate (SDS) polyacrylamide gel] and immunodetection, using rabbit polyclonal antibody against GLUT4 (1:3000; EMD Millipore, Bilerica, MA, USA, #07-1404). The blots were quantified by densitometry (ImageQuant TL, Amersham Biosciences UK Limited), and the densities of the respective lanes, stained by Ponceau, were used for normalization. The results were expressed as arbitrary units, related to mean of the controls, which was set as 1.0.

### Statistical analysis

Statistical analysis was performed using GraphPad Prism 5.0 software (GraphPad Prism, Inc., San Diego, CA, USA). One-way analysis of Variance (ANOVA) with Newman-Keuls post-test for comparison between groups or Kruskal-Wallis test followed by multiple Dunns for non-parametric data were used. A two-way analysis was used to determine weight gain. Unpaired Student *t*-test was utilized to compare C and AGE-albumin regarding AGE levels. Results are expressed as mean ± standard errors of the mean (SEM); a *p*-value < 0.05 was considered statistically significant.

RNA-sequencing data was loaded in R, annotated using BioMart and analyzed using the limma package from Bioconductor. The expression values were adjusted according to the surrogate variables identified by SVA using the function removeBatchEffect from the limma package. Next, one different type of supervised analyses was performed using limma between AGE- and C-albumin. Enrichment for gene ontology (GO) terms for individual comparisons was performed by hypergeometric tests using the GOstats package from Bioconductor (Durinck et al., 2009; Ritchie et al., 2015)^1^.

## Results

**Figure 1:**
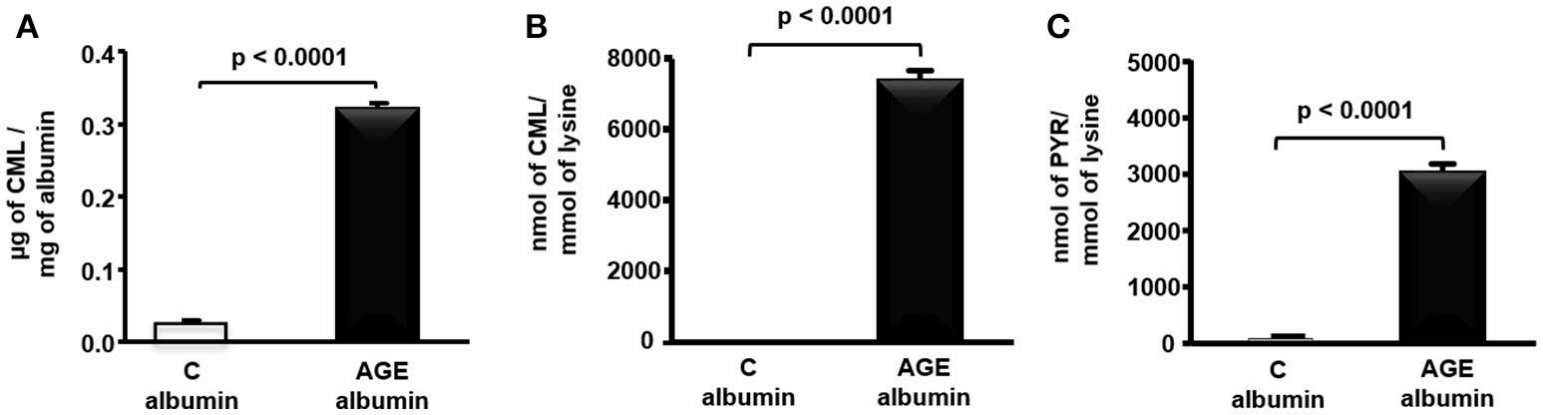
Carboxymethyllysine (CML) and pyrraline (PYR) in control (C) and advanced glycated (AGE) rat albumin. Rat fatty acid free albumin (RSA) was incubated with 10 mM glycolaldehyde (AGE-albumin) or PBS only (C albumin) for 4 days, at 37°C, under sterile conditions. CML content was quantified in albumin by ELISA **(A)** and LC-MS/MS **(B)**, and PYR **(C)**, by LC-MS/MS. Results were compared by the unpaired Student's *t*-test (*n* = 3).

CML determined by ELISA was 12.6 times greater in rat AGE-albumin as compared to C-albumin (Figure 1A). In agreement, a more specific measurement of this AGE by LC-MS/MS, demonstrated greater levels of CML as well as PYR in AGE-albumin in comparison to C samples (Figures 1B,C).

**Figure 2:**
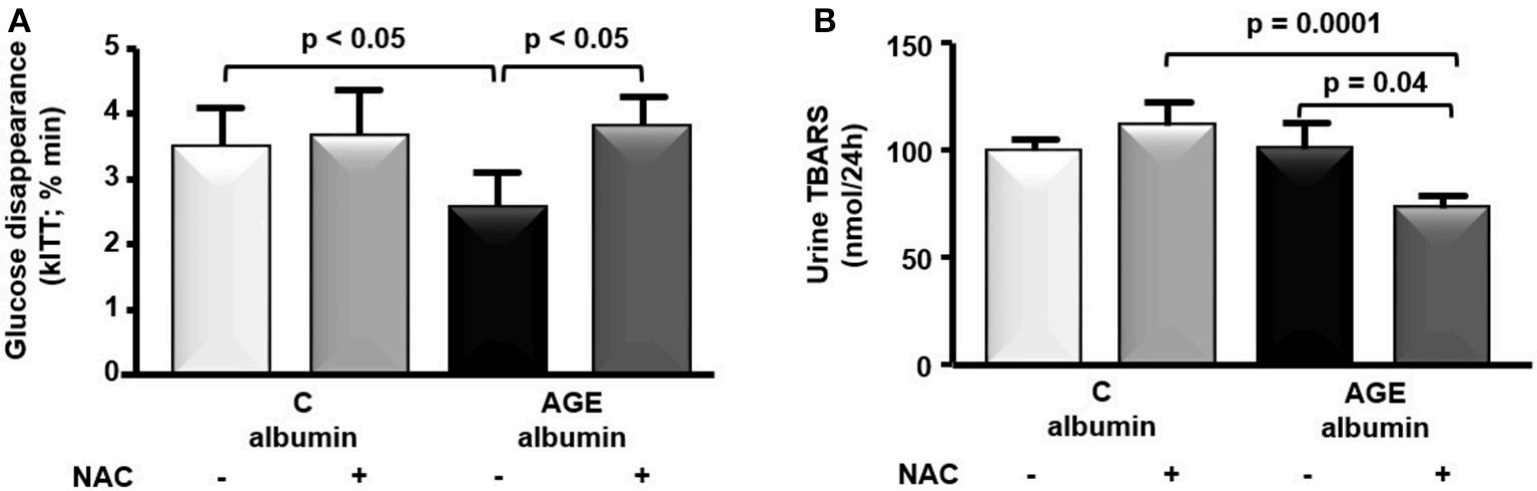
Insulin tolerance test and urine thiobarbituric acid reactive substances (TBARS). **(A)** At the end of the protocol, insulin tolerance test (ITT) was performed in animals treated with C-albumin (*n* = 3), C-albumin + NAC (*n* = 4), AGE- albumin (*n* = 4), and AGE-albumin + NAC (*n* = 4). Blood samples were collected from the tail vein 0, 4, 8, 12, and 16 min after an intravenous injection (penis vein) of regular insulin (100 UI/mL/kg) and glucose disappearance constant (kITT; % min) was determined. **(B)** TBARS (nmol/24 h), markers of lipid peroxidation, were determined in 24-h urine samples at the end of the protocol in C (*n* = 6), C + NAC (*n* = 5), AGE (*n* = 6), and AGE + NAC (*n* = 5) groups. The optical density was read at 534 nm. Results (mean ± SEM) were compared by One-way ANOVA with Newman-Keuls post-test.

Food consumption and rat body weight were similar among groups (Table 1). Plasma lipids (total cholesterol, triglycerides, FFA) glucose, insulin, urea, creatinine, alanine and aspartate aminotransferase levels as well as 24 h-urinary protein excretion and systolic blood pressure were similar in all groups (Table 1). Despite that, a reduced glucose decay rate assessed by the ITT was observed in animals treated with AGE-albumin as compared to C-albumin and this was prevented by NAC treatment (Figure 2A). Urine TBARS, which strongly correlate with plasma lipid peroxidation, were similar in C and AGE-albumin-treated animals, but were reduced in AGE + NAC as compared to AGE and C + NAC rats (Figure 2B).

**Table 1:** Biochemical parameters of animals treated with C or AGE-albumin, concomitantly or not NAC, at the end of protocol.

	**C albumin (*n* = 8)**	**C + NAC albumin (*n* = 7)**	**AGE albumin (*n* = 8)**	**AGE + NAC albumin (*n* = 7)**
Food intake (g)	30.3 (23.5–35)	30 (24–35)	31 (24–33)	31.4 (26–34)
Body weight (g)	501 ± 24	511 ± 17	506 ± 17	520 ± 15
SBP (mmHg)	121 (105–125)	121 (95–128)	120 (93–144)	117 (102–142)
TC (mg/dL)	69 (61–77)	73 (63–97)	69 (49–101)	71 (51–83)
TG (mg/dL)	41 (27–80)	41 (25–70)	36 (29–68)	26 (23–42)
Glucose (mg/dL)	114 ± 5.0	115 ± 3.0	112 ± 3.8	117 ± 4.2
Insulin (ng/dL)	1.2 (0.3–3.0)	1.8 (1.1–2.5)	1.3 (0.9–2.9)	1.4 (0.9–2.9)
FFA (mg/dL)	0.3 (0.1–0.5)	0.4 (0.1–0.6)	0.3 (0.1–0.4)	0.4 (0.1–0.4)
Urea (mg/dL)	44 ± 2.7	43 ± 1.4	49 ± 3.5	44 ± 2.3
Creatinine (mg/dL)	0.3 ± 0.01	0.2 ± 0.05	0.3 ± 0.05	0.3 ± 0.05
Proteinuria (mg/24 h)	8.0 ± 0.8	9.0 ± 1.6	9.0 ± 1.1	9.0 ± 1.5
ALT (U/L)	38 (28–67)	30 (26–54)	42 (33–55)	35 (30–65)
AST (U/L)	88 (65–124)	96 (61–123)	84 (63–146)	107 (57–133)

*Results expressed as mean ± SEM or median (range) were compared by One-way ANOVA with Newman-Keuls post-test or Kruskal-Wallis test followed by multiple Dunns for non-parametric data. A two-way analysis was used to determine body weight gain. SBP, systolic bood pressure; TC, Total cholesterol; TG, triglicerydes; FFA, Free fatty acid; ALT, Alanine aminotransferase; AST, Aspartate aminotransferase*.

**Figure 3:**
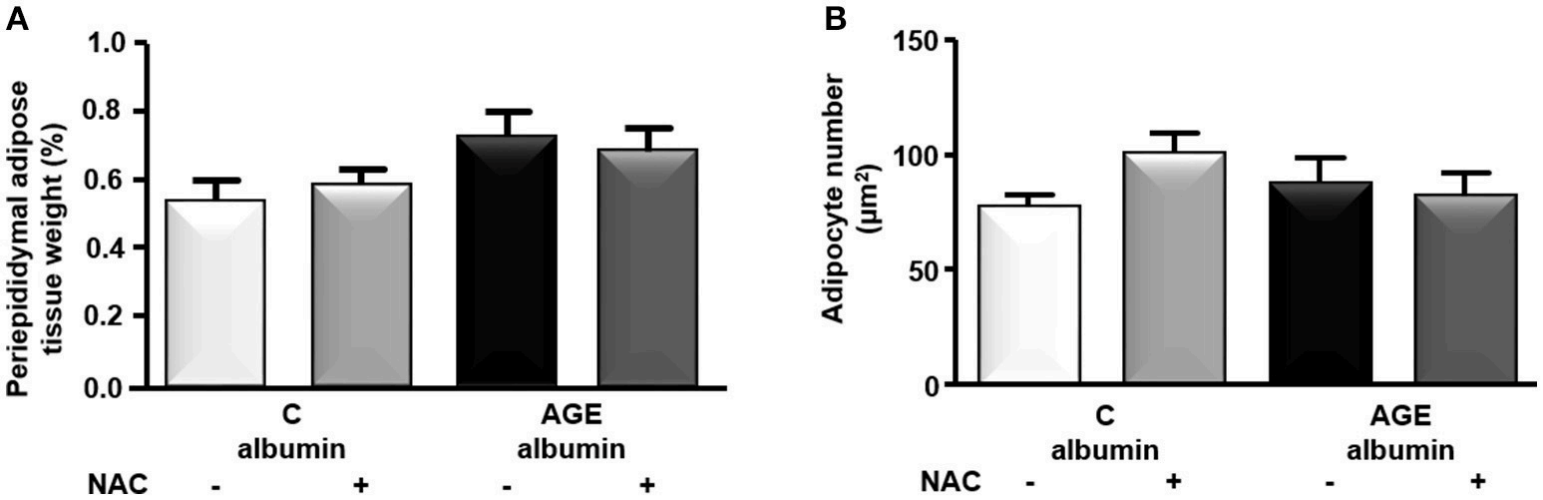
Periepididymal adipose tissue morphology. Periepididymal fat from animals treated with C-albumin (*n* = 8), C-albumin + NAC (*n* = 7), AGE- albumin (*n* = 8), and AGE-albumin + NAC (*n* = 7) was excised and weighed to calculate its relative mass **(A)**. Adipocyte number per μm^2^ **(B)** was estimated by stereological method (counting-points). Results (mean ± SEM) were compared by One-way ANOVA with Newman-Keuls post-test.

**Figure 4:**
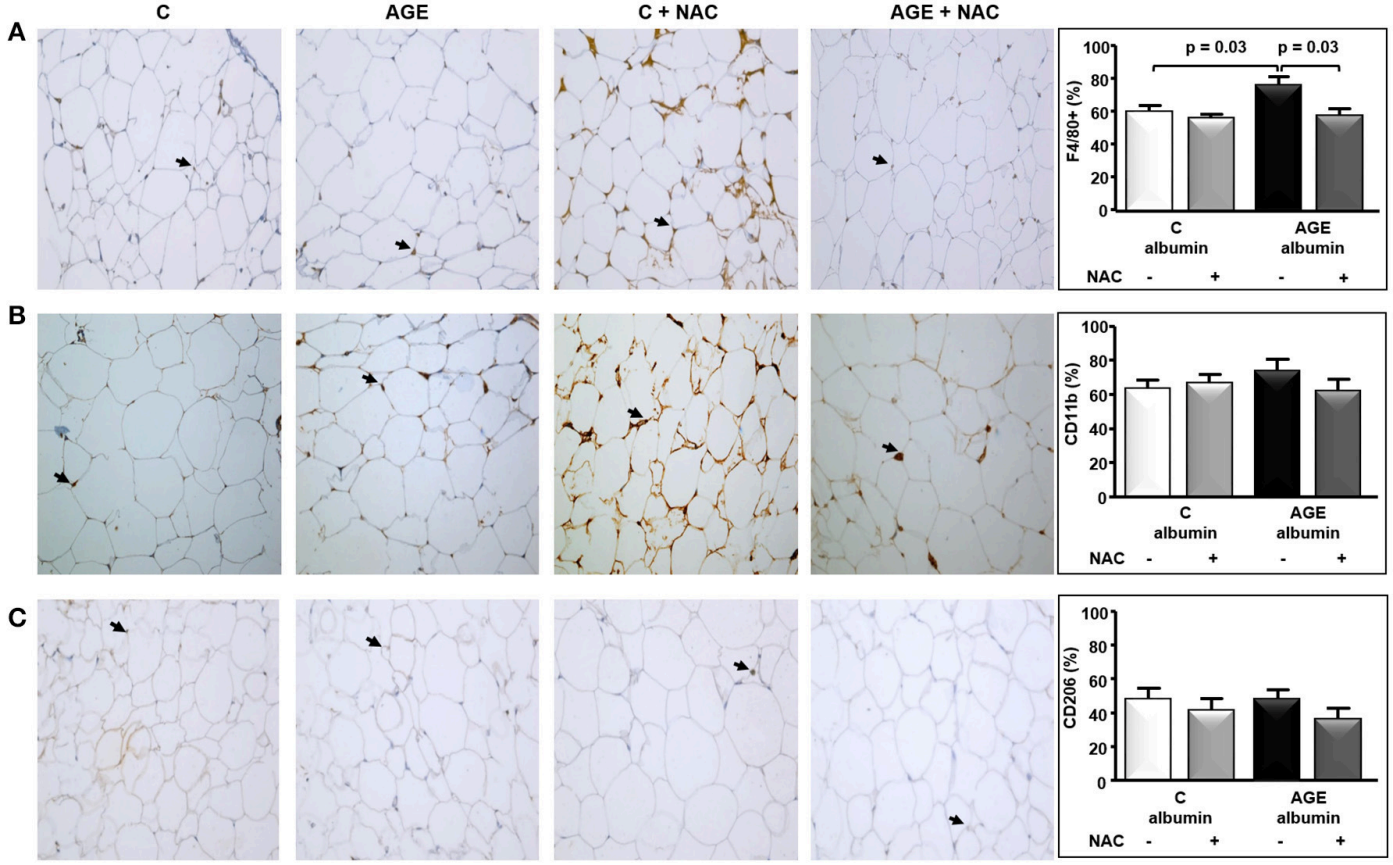
Immunostaining for F4/80, CD11b, and CD206 in periepididymal adipose tissue. Periepididymal adipose tissue sections from animals treated with C-albumin (*n* = 8), C-albumin + NAC (*n* = 7), AGE-albumin (*n* = 8) and AGE-albumin + NAC (*n* = 7) were evaluated by immunohistochemistry. **(A)** Representative images of histological sections; black arrows indicate positive cells (macrophage) stained with antibody against F4/80 (Abcam 1:100); 400 X magnification. **(B)** Representative images of histological sections; black arrows indicate positive cells stained with antibody against CD11b, marker of M1-macrophage (Abcam 1:200); 400 X magnification. **(C)** Representative images of histological sections; black arrows indicate positive cells stained with antibody against CD206, marker of M2-macrophage (Abcam 1:1250) (400 X magnification). Representative graphics were obtained after stereological analysis. Results (mean ± SEM) were compared by One-way ANOVA with Newman-Keuls post-test.

The periepididymal adipose tissue relative weight and adipocyte number were similar among groups (Figures 3A,B). The number of F4/80-positive (F4/80+) macrophages was 1.3 times higher in AGE-albumin-treated animals than in C-albumin and was reduced in AGE + NAC (Figure 4A). Nonetheless, we did not find changes in the amounts of CD11b (a marker of M1 phenotype) (Figure 4B) and of CD206 (a marker of M2 phenotype) (Figure 4C).

**Figure 5:**
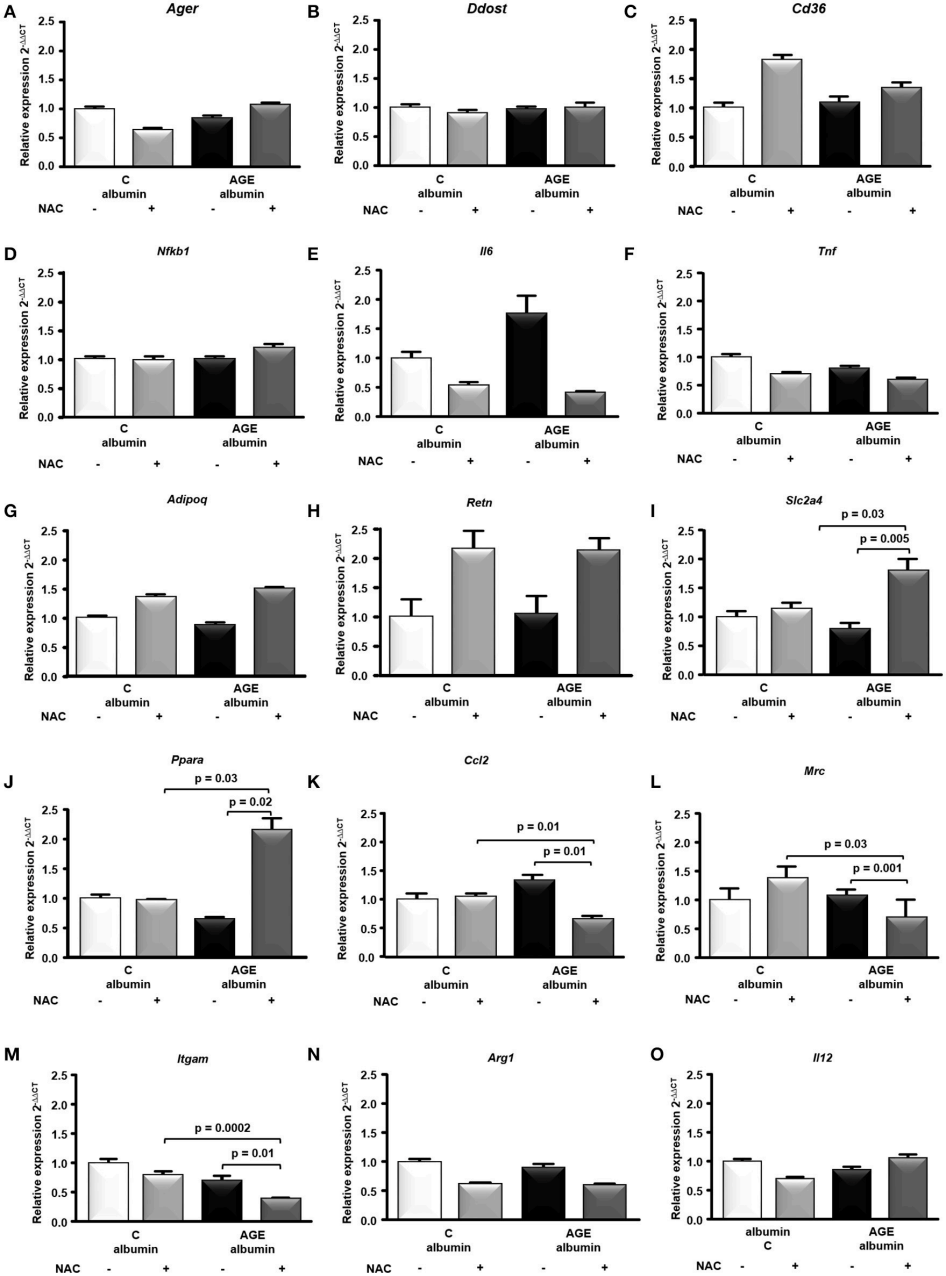
Gene expression in periepididymal adipose tissue. *Ager* **(A)**, *Ddost* **(B)**, *Cd36* **(C)**, *Nfkb1* **(D)**, *Il6* **(E)**, *Tnf* **(F)** *Adipoq* **(G)**, *Retn* **(H)**, *Slc2a4* **(I)**, *Ppara* **(J)**, *Ccl2* **(K)**, *Mrc* **(L)**, *Itgam* **(M)**, *Arg1* **(N)**, *Il12* **(O)** mRNA were evaluated in periepididymal adipose tissue from animals treated with C-albumin (*n* = 8), C-albumin + NAC (*n* = 7), AGE-albumin (*n* = 8) and AGE-albumin + NAC (*n* = 7), by real time quantitative PCR (RT-qPCR; Applied Biosystems, Foster City, CA, USA). The relative expression of each gene was normalized to the housekeeping *Hprt1* gene and relative quantification analysis was performed using the comparative cycle threshold (Ct) (2−^ΔΔCt^) method. Results (mean ± SEM) were compared by One-way ANOVA with Newman-Keuls post-test or Kruskal-Wallis followed by multiple Dunns test.

The expression of genes related to AGE signaling in adipocytes and the major adipokines, *Ager, Ddost, Cd36, Nfkb1, Il6, Tnf*, *Adipoq*, and *Retn* mRNA were similar among groups (Figures 5A–H). On the other hand, genes related to glucose homeostasis and macrophage differentiation, such as *Slc2a4* and *Ppara*, were increased while *Ccl2, Mrc* (M2 marker) and *Itgam* were decreased in AGE + NAC in comparison to AGE and C + NAC animals (Figures 5I–M). *Arg1* (M2 marker) and *Il12* mRNA were not different among groups (Figures 5N,O).

**Figure 6:**
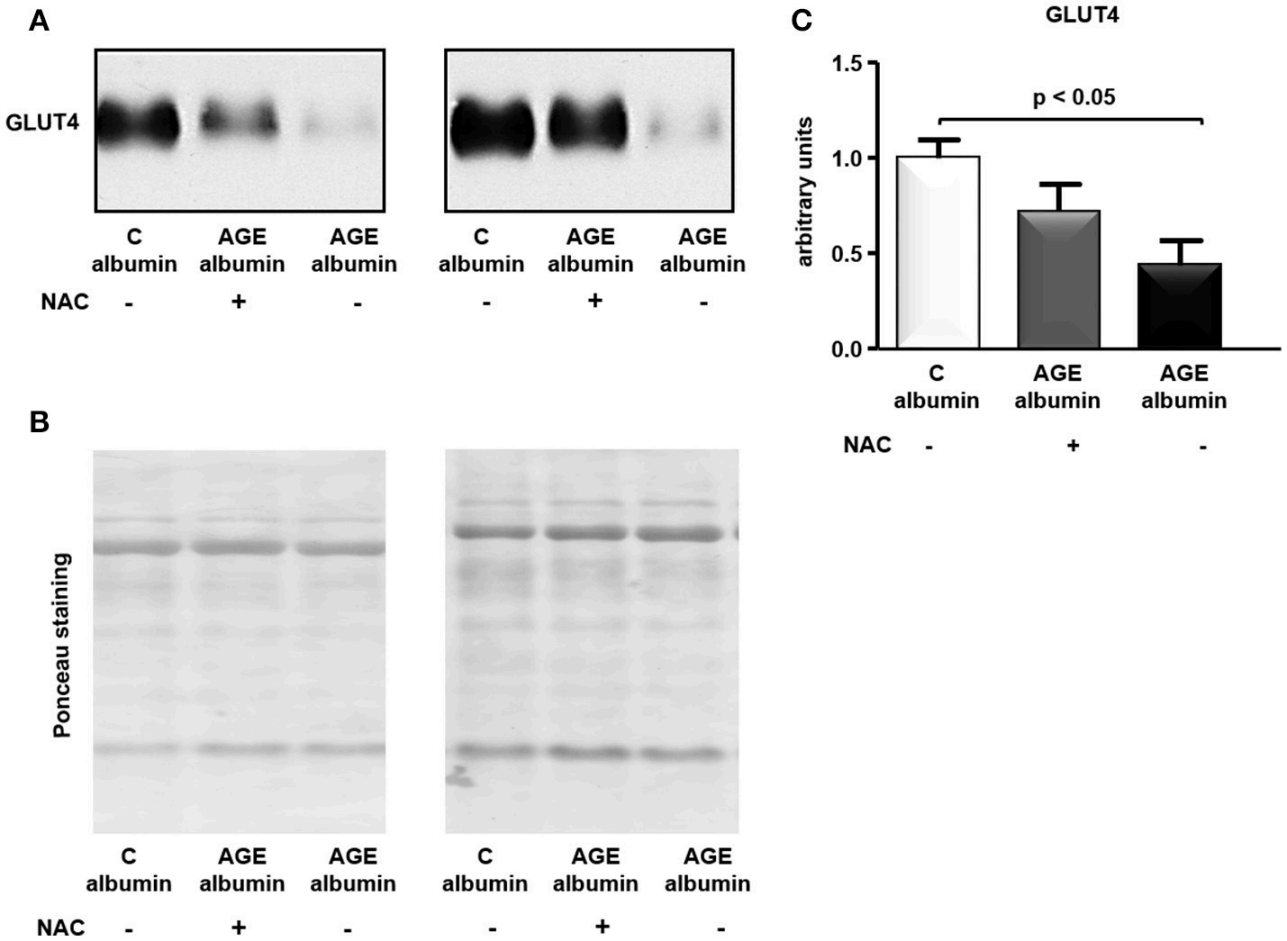
GLUT4 protein level in periepididymal adipose tissue. GLUT4 was determined in adipose tissue from animals treated with C-albumin (*n* = 4), AGE-albumin (*n* = 6), and AGE-albumin + NAC (*n* = 6); equal amounts of cellular protein were applied into a 10% T SDS-polyacrylamide gel and then submitted to immunoblot using rabbit polyclonal antibody against GLUT4 (1:3000; EMD Millipore, Bilerica, MA, USA, #07-1404). The blots were quantified by densitometry (ImageQuant TL, Amersham Biosciences UK Limited) (representative images, **A**). The Ponceau-stained nitrocellulose membrane was used as loading control **(B)**; after Ponceau normalization results were expressed as arbitrary units considering the mean of the control as 1 in each gel **(C)**.

The total amount of GLUT4 in adipose tissue was assessed by immunoblot. As shown in Figures 6A–C, treatment with AGE-albumin reduced GLUT4 in comparison to C-albumin, which was not completely prevented in animals treated with AGE-albumin plus NAC.

**Figure 7:**
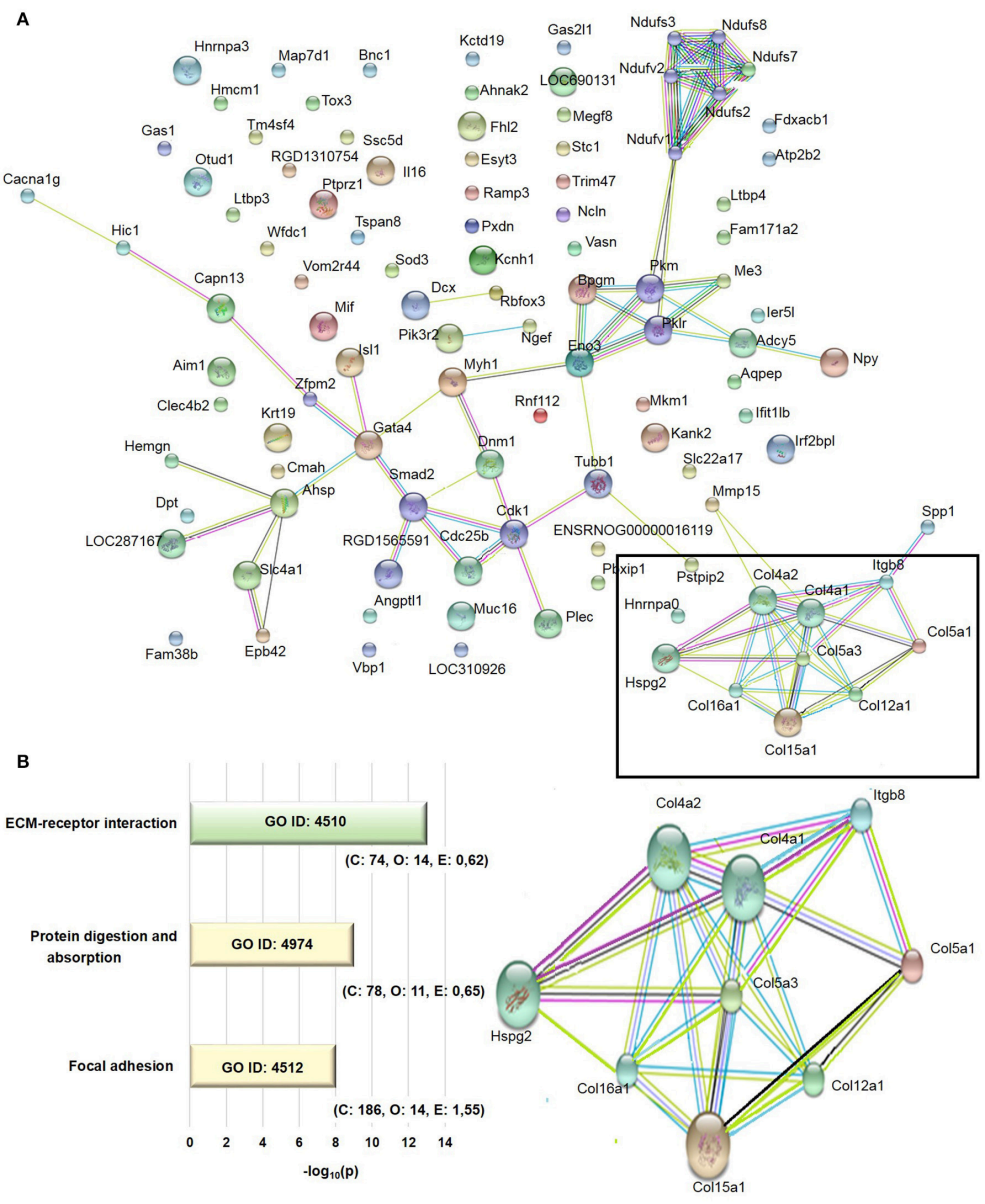
Transcriptome analysis of periepididymal adipose tissue. **(A)** Identification of network interactions of differentially expressed genes between C and AGE-albumin-treated animals. Bioinformatic analysis of RNA-seq data were performed by DeSeq2 Version 3.5, GO, GSEA and compared to STRING 9.0, using an interaction score 0.400 - medium confidence. Signal *p*-value > 0.1, fold change <0.04, micro-RNA and pseudogenes were excluded. **(B)** The extracelullar matrix-receptor interaction (ECM-receptor interaction), protein digestion and absorption and focal adhesion were obtained by gene ontology (GO) enrichment analysis. Number of reference genes in the category (C), number of genes in the set and also in the category (O) and the expected number (E) in the category were observed after *p*-value cutoff of <0.01 and fold-change >0.4 adjusted. Adjusted p (AdjP) were converted in –log10 with variation between 8 and 13.

**Figure 8:**
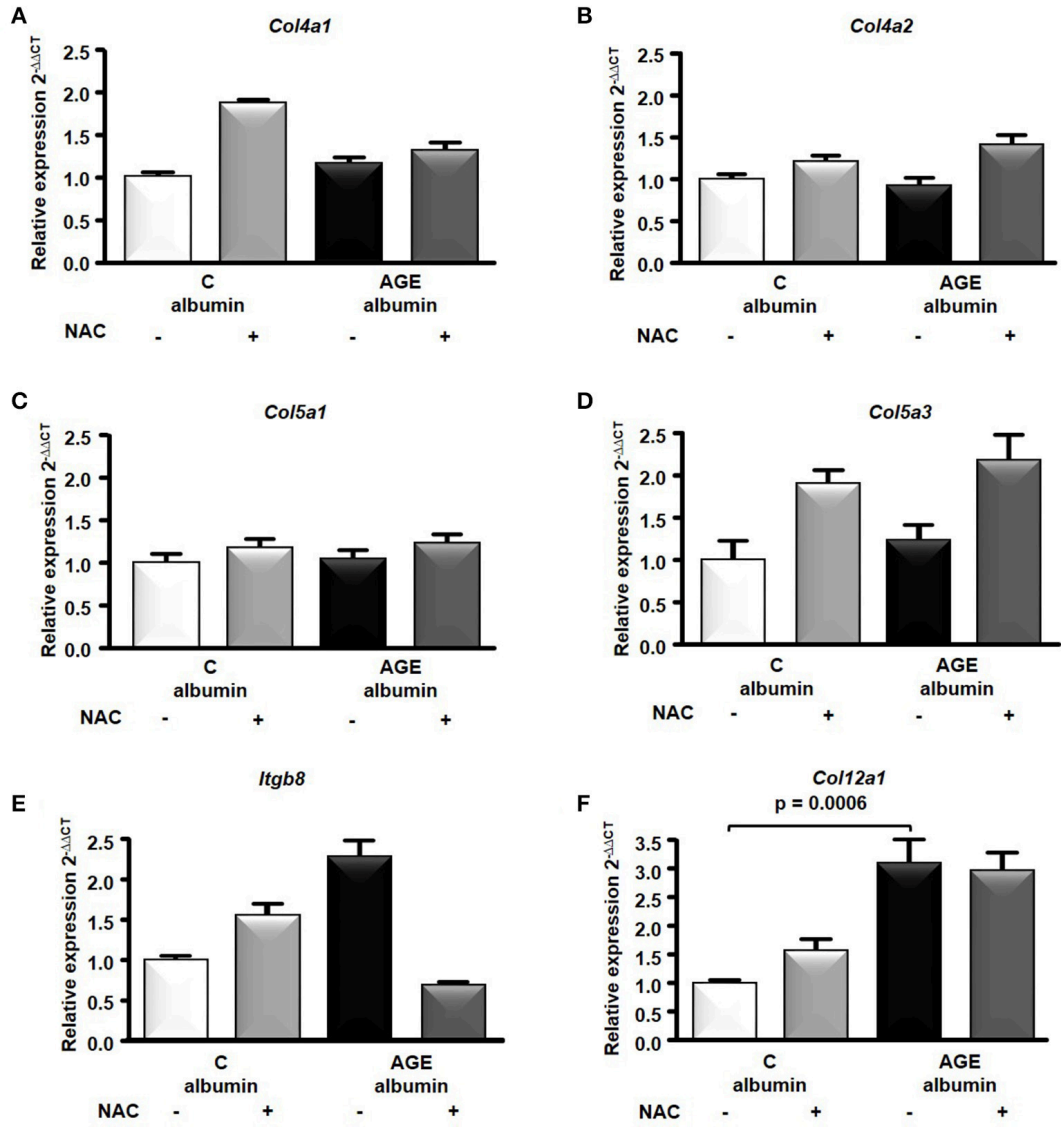
RNA-seq of the periepididymal adipose tissue. *Col4a*1 **(A)**, *Col4a2* **(B)**, *Col5a1* **(C)**, *Col5a3* **(D)**, *Itgb8* **(E)**, *Col12a1***(F)** mRNA were validated in periepididymal adipose tissue from animals treated with C (*n* = 7) or AGE- albumin (*n* = 8) by real time quantitative PCR (RT-qPCR; Applied Biosystems, Foster City, CA, USA). The relative expression of each gene was normalized to the housekeeping *Hprt1* and relative quantification analysis was performed using the comparative cycle threshold (Ct) (2−^ΔΔCt^) method. Results (mean ± SEM) were compared by One-way ANOVA with Newman-Keuls post-test or Kruskal-Wallis followed by multiple Dunns test.

The transcriptome analysis revealed 18,810 differentially expressed genes, which were stringency-adjusted to *p*-value, fold change cutoff and Bonferroni corrections. After that, ~100 genes were obtained and analyzed by enrichment analysis (GO) and by STRING, where an enriched functional category related to matrix components, primarily represented by the collagen and integrin families, was identified (Table S1). Gene and protein interaction map is depicted in Figures 7A,B. RT-qPCR was utilized to validate differences demonstrated in the transcriptome analysis. The expression of *Col4a1, Col4a2, Col5a1, Col5a3*, and *Itgb8* were similar among groups (Figures 8A–E). In contrast, AGE-albumin increased *Col12a1* mRNA levels as compared to C-albumin and NAC did not change this expression level (Figure 8F).

## Discussion

In an attempt to understand the influence of AGEs in adipocyte-triggered inflammation and in glucose homeostasis, independently of other metabolic pathways that are present in DM, we looked at the histological aspects of the periepididymal adipose tissue, macrophage infiltration and differentiation and gene expression profile by chronically treating healthy Wistar rats with AGE-albumin. Considering the role of the antioxidant N-acetylcysteine in the prevention of intracellular damage elicited by AGEs (Machado et al., 2014) we also analyzed the role of this compound in animals treated with C or AGE-albumin.

A greater amount of AGEs was found in rat albumin exposed *in vitro* to glycolaldehyde. This oxoaldehyde leads to a rapid and irreversible change of albumin by CML and PYR that in fact were highly elevated in these samples according to MS/MS analysis and ELISA. CML is elevated in plasma and tissues of DM patients and is a prevalent AGE in heat-processed foods. In addition, CML is a classic ligand for RAGE triggering the transactivation of inflammatory genes by the activation of NFKB (Kislinger et al., 1999).

It is conceivable that in obese animals an enhanced macrophage infiltration is observed in hyperplastic/hypertrophied adipose tissue that relates to a more pronounced differentiation into the M1 classically activated inflammatory cell phenotype that accompanies insulin resistance (Lumeng et al., 2007, 2008). Nonetheless, the specific determination of M1 or M2 phenotypes *in vivo* is limited due to a broad spectrum of macrophage polarization subsets (Martinez and Gordon, 2014; Murray et al., 2014). In the present investigation, after 90 consecutive days of treatment with C or AGE-albumin alone or with NAC in the drinking water, we did not find alterations in rat body weight or periepididymal adipose tissue weight and adipocyte number. Despite that, we found an increased immunostaining for F4/80 macrophages in adipose tissue of AGE-albumin-treated animals. Even though we were unable to differentiate between M1 and M2 phenotypes by utilizing immunostaining and gene expression (RT-qPCR) techniques, the long-term treatment with AGEs triggered adipocyte tissue inflammation by recruiting more macrophages to fat depots even in a non-obese animal with a normal plasma lipid profile.

FFAs are important mediators of monocyte recruitment to adipocytes (Lumeng et al., 2007) and impair insulin signaling although they were normal in our AGE-treated rat. In addition, the monocyte chemo-attractant protein 1 (MCP-1) is the major chemokine produced by stressed adipocytes that induces macrophage infiltration. While AGE-albumin by itself did not elicit a higher expression of *Ccl2* gene, NAC reduced its expression, which accords with its ability in reducing macrophage infiltration and increasing insulin sensitivity.

We did not detect differences elicited by AGE-albumin in the expression of genes analyzed in the adipose tissue by RT-qPCR. Only in the presence of AGE, NAC was able to increase the expression of *Slc2a4*. On the other hand, the amount of GLUT4 protein in adipose tissue was reduced by AGE-albumin in comparison to C-albumin, and NAC seems to partially prevent that. The discrepancies between mRNA expression and GLUT4 protein level may be related to post-transcriptional mechanisms that regulate the final protein content of this glucose transporter as previously reported (Muñoz et al., 1996; Seraphim et al., 2007).

PPAR alpha whose gene was increased in NAC+AGE group is mostly known for its ability to induce fatty acid oxidation, minimizing the deleterious effects of those lipid derivatives on the insulin receptor signaling via IRS1. In addition, PPAR alpha cooperatively activates the *Slc2a4* gene, improving glucose uptake (Yao et al., 2012), and mediates the transrepression of inflammatory genes that also contribute to the amelioration of insulin sensitivity (Rakhshandehroo et al., 2010). NAC was able to decrease the expression of *Ccl2* that may contribute to the reduction in macrophage infiltration and inflammation that modulates insulin sensitivity. Due to limited protein availability we were not able to perform immunoblotting for PPAR alpha and MCP-1.

In macrophages, Okuda et al (Okuda et al., 2012) demonstrated that AGE-albumin by itself did not increase inflammation; but rather primed those cells to the inflammatory stimulus by lipopolysaccharides or calgranulins that impairs the apo A-I-mediated cholesterol removal in naive cells. In another study, AGE-albumin chronically infused into the peritoneal cavity of non-diabetic dyslipidemic mice increased aortic lipid infiltration, CML, RAGE, 4-hydroxynonenal, and inflammatory cytokines. Demonstrating a resemblance to our findings in the present study, those effects were independent of changes in plasma lipids, glucose and blood pressure (Gomes et al., 2016), pointing to a direct role of AGEs in the development of chronic degenerative disease such as atherosclerosis even in the absence of DM.

Animals treated with AGE-albumin developed an insulin resistant state according to the reduced glucose decay rate observed in the ITT. It had previously been demonstrated *in vitro* that AGEs induce ROS generation, while reducing insulin signaling and glucose uptake in cultured adipocytes (Unoki et al., 2007; Wu et al., 2011). In addition, AGE-albumin induced protein carbonylation in 3T3-L1 adipocytes, which is related to inhibition of the proteasomal system and dysregulation in cellular redox balance (Singh et al., 2007). These events seem to depend on chronic stimulation by AGE and can be blocked by the immunological suppression of RAGE and by antioxidants such as NAC (Ramasamy et al., 2007). In the present study, we did not analyze the insulin-signaling pathway, but the final effector of the insulin signal that mediates glucose uptake, GLUT4, was reduced by AGE-albumin. It is worth considering that in our experimental model, AGEs may have triggered adipose tissue inflammation and that the absence of changes in FFA and adiposity may have masked more dramatic effects in this tissue that can be attained over the long-term.

The insulin resistance observed in rats chronically treated with AGE-albumin may be more related to skeletal muscle damage. In this sense, recent observations from our group in these animals demonstrated that AGE-albumin diminished *Ddost* and *Slc2a4* mRNA without changes in GLUT4 protein level. Besides, AGE-albumin increased nuclear NFKB and the expression of endoplasmic reticulum stress markers (Grp78/94 chaperones) (unpublished data). Chiu et al. (2016) also showed that AGE reduced insulin signaling, namely PI3K and AKT phosphorylation in cultured muscle cells. Coughlan et al. (2011) in a similar protocol demonstrated that AGE-albumin induced profound changes in islets' cytoarchitecture that make rats prone to insulin secretion failure. Thus, AGEs seems to act as hit-model, inducing insulin resistance independently of changes in body weight and circulating FFA. The animals' response to chronic AGE treatment may be organ-specific and possibly conditioned to the flux of AGE-albumin to the interstitium as well as to the balance between RAGE and AGER-1 and consequently anti and pro oxidant systems. These variables should be evaluated in future studies.

Heat-processed foods, especially those containing high amounts of fat, are the major components of modern diets and contribute to the body pool of AGEs (Uribarri et al., 2010). High AGE-containing diets induce insulin resistance; on the other side, AGE restriction in food ameliorates insulin signaling, and increases sirtuin-1 expression and AGER-1 that mitigate deleterious gene expression elicited by the AGE/RAGE axis (Cai et al., 2012; Vlassara et al., 2016).

In an exploratory analysis, trying to find new biomarkers and cell metabolism modulators we evaluated by RNA-seq the differential expression of genes in adipocytes. Matrix components mainly related to the collagen family were differentially expressed in adipose tissue from AGE-treated rats. After RT-qPCR validation, only *Col12a1* differed significantly between C and AGE. In association with fibrils, collagen XII confers bone and muscle integrity (Chiquet et al., 2014). Although collagen has been reported as the major protein modified by advanced glycation due to its long half-life, the implication of *Col12a1* overexpression in adipocytes from AGE-treated animals should be further investigated.

Interestingly, insulin resistance prevented by NAC, which was also able to reduce lipid peroxidation (TBARS), adipose tissue macrophages (total number and M1 and M2 markers, respectively, *Itgam* and *Mrc*) and to enhance *Slc2a4, Ppara* in adipose tissue. It had been previously shown that orally administered NAC (1.5 g/Kg/day) enhanced AKT and STAT3 phosphorylation in streptozotocin-induced DM rats (Wang et al., 2013). Trewin et al. (2015) showed that the intravenous infusion of NAC increased insulin sensitivity in skeletal muscle independently of AKT. In addition, it was recently demonstrated that NAC reduced plasma TBARS, total AGE and pentosidine and diminished the deleterious effects of serum AGE-albumin, isolated from chronic kidney disease rats in macrophage endoplasmic reticulum stress (Machado et al., 2014). In rats chronically injected with AGE-albumin, NAC was able to reduce the enhanced infiltration of macrophages (CD68-positive cells) in tubule-interstitium. In addition, NAC treatment significantly decreased ROS generation and the expression of *Ager, Ren, Col4a1*, and the *Bax* to *Bcl2* ratio induced by AGE-albumin in healthy rats (Thieme et al., 2016). By inducing glutathione synthesis, orally administered NAC is effective in improving antioxidant defenses with a favorable safety profile (Machado et al., 2014; Thieme et al., 2016). Besides, NAC is an important source of sulfhydryl groups and favors quenching and detoxification of reactive oxygen species (Dhouib et al., 2016).

In conclusion, chronic administration of AGE-albumin in healthy rats sensitizes the adipose tissue to inflammation due to macrophage infiltration and reduces GLUT4, contributing to an insulin resistant state. NAC antagonizes the effects of AGE-albumin and, by itself, has beneficial effects in macrophage differentiation on adipose tissue, inflammatory response, lipid peroxidation and insulin sensitivity. Our data reinforce the idea that AGEs have detrimental effect that are independent of the presence of hyperglycemia and that NAC may be a useful tool in the prevention of AGE actions on the development of insulin resistance and complications of DM.

## Author contributions

KdS: Performed the experiments, statistics and wrote the manuscript; NF: Conducted animal treatment and biochemical analysis; PP: Helped in mRNA analysis; DG: Helped in surgical procedures; KT: Helped in mRNA analysis; LO: Performed LC/MS analysis; RI: Performed ELISA analysis; VF: Helped in the transcriptome analysis; MS: Performed TBARS analysis; WT: Supervised the immunohistochemistry analysis; SM: Supervised the transcriptome analysis; TW: kindly provided the chromatography standards for AGE analysis; MB: kindly provided the chromatography standards for AGE analysis; RP: Helped in the LC/MS analysis; KR: Supervised LC/MS analysis; MO: Performed ITT and immunoblotting; SC: Supervised animal care and surgical procedures; UM: Helped in data interpretation and manuscript revision; MC: Helped in data interpretation and manuscript revision; MP: Designed the study, interpreted the results and wrote the manuscript.

### Conflict of interest statement

The authors declare that the research was conducted in the absence of any commercial or financial relationships that could be construed as a potential conflict of interest. The reviewer DV and handling Editor declared their shared affiliation.

## Footnotes

^1^*R: The R Project for Statistical Computing*. Available online at: https://www.r-project.org/ (Accessed: Feb 5, 2017).

## Supplementary material

Table S1 Transcriptome analysis of periepididymal tissue. 18,810 differentially expressed genes were identified in the periepididymal adipose tissue of healthy rats treated with C or AGE-albumin. After corrections, ~100 genes were obtained and an enriched category related to matrix components was identified.
