# Supplementary material for: N-acetylcysteine Counteracts Adipose Tissue Macrophage Infiltration and Insulin Resistance Elicited by Advanced Glycated Albumin in Healthy Rats
Source: Front Physiol. 2017 Sep 22;8:723. doi: 10.3389/fphys.2017.00723 (PMC5616024; doi:10.3389/fphys.2017.00723)
Supplement: Table S1 — Transcriptome analysis of periepididymal tissue. 18,810 differentially expressed genes were identified in the periepididymal adipose tissue of healthy rats treated with C or AGE-albumin. After corrections, ~100 genes were obtained and an enriched category related to matrix components was identified. [file Table1.pdf]

Gene	Description	entrezgene	FoldChange
Trim47	tripartite motif-containing 47 [Source:RGD Symbol;Acc:1597317]	690374	0,727520612
Kctd19	potassium channel tetramerization domain containing 19 [Source:RGD Symbol;Acc:1561326]	291965	-0,945481679
Spp1	secreted phosphoprotein 1 [Source:RGD Symbol;Acc:3752]	25353	0,955204054
Dcx	doublecortin [Source:RGD Symbol;Acc:620670]	84394	0,923893852
Muc16	mucin 16, cell surface associated [Source:RGD Symbol;Acc:1308422]		-0,757416294
Epb42	erythrocyte membrane protein band 4.2 [Source:RGD Symbol;Acc:1305306]	362202	-0,935816058
Tubb1	tubulin, beta 1 class VI [Source:RGD Symbol;Acc:1589557]	679312	-0,903593932
Hemgn	hemogen [Source:RGD Symbol;Acc:621569]	113882	-0,925784006
Ier5l	immediate early response 5-like [Source:RGD Symbol;Acc:1561058]	499772	0,883679475
Ramp3	receptor (G protein-coupled) activity modifying protein 3 [Source:RGD Symbol;Acc:61873]	56820	-0,717494236
Npy	neuropeptide Y [Source:RGD Symbol;Acc:3197]	24604	-0,901534476
LOC100912228	pro-neuropeptide Y-like [Source:RGD Symbol;Acc:6487865]	100912228	-0,901534476
Col5a1	collagen, type V, alpha 1 [Source:RGD Symbol;Acc:70920]	85490	0,648694913
LOC100909555	uncharacterized LOC100909555 [Source:RGD Symbol;Acc:6503001]		0,810806155
Col15a1	collagen, type XV, alpha 1 [Source:RGD Symbol;Acc:1310820]		0,559822963
Hmcn1	hemicentin 1 [Source:RGD Symbol;Acc:1564772]	289094	0,64972166
LOC100909752	collagen alpha-1(XV) chain-like [Source:RGD Symbol;Acc:6502064]	100909752	0,551823073
Hist2h2aa2	histone cluster 2, H2aa2 [Source:RGD Symbol;Acc:1584037]	365877	0,691526462
Col16a1	collagen, type XVI, alpha 1 [Source:RGD Symbol;Acc:1310982]	366474	0,398118558
Megf8	multiple EGF-like-domains 8 [Source:RGD Symbol;Acc:621190]	114029	0,428937522
Fhl2	four and a half LIM domains 2 [Source:RGD Symbol;Acc:61963]	63839	0,784498475
Col4a2	collagen, type IV, alpha 2 [Source:RGD Symbol;Acc:1308085]	306628	0,442874163
Dpt	dermatopontin [Source:RGD Symbol;Acc:1307176]	289178	0,569180566
Col5a3	collagen, type V, alpha 3 [Source:RGD Symbol;Acc:70922]	60379	0,53640596
Mkrn1	makorin ring finger protein 1 [Source:RGD Symbol;Acc:1303251]	296988	-0,551768763
Map7d1	MAP7 domain containing 1 [Source:RGD Symbol;Acc:1597986]	681287	0,464933925
Ahsp	alpha hemoglobin stabilizing protein [Source:RGD Symbol;Acc:1306725]	293522	-0,796509874
Rbfox3	RNA binding protein, fox-1 homolog (C. elegans) 3 [Source:RGD Symbol;Acc:1560070]	287847	-0,751081059
Cmah	cytidine monophospho-N-acetylneuraminic acid hydroxylase [Source:RGD Symbol;Acc:1311930]	361245	-0,521764024
Itgb8	integrin, beta 8 [Source:RGD Symbol;Acc:1311374]	362800	-0,724462249
LOC100362814	hypothetical protein LOC100362814 [Source:RGD Symbol;Acc:2323964]		0,783017716
Col28a1	collagen, type XXVIII, alpha 1 [Source:RGD Symbol;Acc:1564680]	312115	-0,589435097
AABR07073181.1	Protein Hspg2 [Source:UniProtKB/TrEMBL;Acc:F1LTJ5]		0,517692107
Lvrn	laeverin [Source:RGD Symbol;Acc:1562779]	502160	-0,627548327

Piezo2	piezo-type mechanosensitive ion channel component 2 [Source:RGD Symbol;Acc:1582973]	307380	0,747691866
Ahnak2	AHNAK nucleoprotein 2 [Source:RGD Symbol;Acc:1309696]		0,444006936
Il16	interleukin 16 [Source:RGD Symbol;Acc:1583872]	116996	-0,543040326
Dnm1	dynamin 1 [Source:RGD Symbol;Acc:71096]	140694	0,569118651
Plec	plectin [Source:RGD Symbol;Acc:621649]	64204	0,451303918
Tm4sf4	transmembrane 4 L six family member 4 [Source:RGD Symbol;Acc:621784]	116467	-0,748809456
Vbp1	von Hippel-Lindau binding protein 1 [Source:RGD Symbol;Acc:1590535]	681825	0,464823705
Bnc1	basonuclin 1 [Source:RGD Symbol;Acc:1309042]	365299	-0,667546663
Cacna1g	calcium channel, voltage-dependent, T type, alpha 1G subunit [Source:RGD Symbol;Acc:68942]	29717	0,579655572
Rnf112	ring finger protein 112 [Source:RGD Symbol;Acc:3986]	24916	-0,704390037
Fam171a2	family with sequence similarity 171, member A2 [Source:RGD Symbol;Acc:1307966]	100361389	0,630115406
Myh1	myosin, heavy chain 1, skeletal muscle, adult [Source:RGD Symbol;Acc:735061]	252942	-0,558220818
Irf2bpl	interferon regulatory factor 2 binding protein-like [Source:RGD Symbol;Acc:1310994]	314329	0,62823828
Col4a1	collagen, type IV, alpha 1 [Source:RGD Symbol;Acc:1307148]	290905	0,440377563
LOC287167	globin, alpha [Source:RGD Symbol;Acc:1359209]	287167	-0,723523174
Tspan8	tetraspanin 8 [Source:RGD Symbol;Acc:621783]	171048	-0,640585043
LOC100912512	ephexin-1-like [Source:RGD Symbol;Acc:6486748]	246217	0,710116326
Tox3	TOX high mobility group box family member 3 [Source:RGD Symbol;Acc:1311529]	291908	0,69455801
Ltbp3	latent transforming growth factor beta binding protein 3 [Source:RGD Symbol;Acc:62057]	83838	0,380856728
Pbxip1	pre-B-cell leukemia homeobox interacting protein 1 [Source:RGD Symbol;Acc:1305180]	310644	0,370316977
Esyt3	extended synaptotagmin-like protein 3 [Source:RGD Symbol;Acc:1561304]	363120	-0,528703949
Hnrnpa0	heterogeneous nuclear ribonucleoprotein A0 [Source:RGD Symbol;Acc:1563684]	681410	0,691512577
Capn13	calpain 13 [Source:RGD Symbol;Acc:1562682]	362701	-0,696838555
Hist2h2aa2	Histone H2A [Source:UniProtKB/TrEMBL;Acc:K7S2S2]	365877	0,592478912
Adcy5	adenylate cyclase 5 [Source:RGD Symbol;Acc:71014]	64532	0,519073002
Stc1	stanniocalcin 1 [Source:RGD Symbol;Acc:621776]	81801	-0,607064419
Fzd7	frizzled class receptor 7 [Source:RGD Symbol;Acc:2321905]	100360552	0,443788826
Angptl1	angiopoietin-like 1 [Source:RGD Symbol;Acc:1598128]	679942	0,691582423
Me3	malic enzyme 3, NADP(+)-dependent, mitochondrial [Source:RGD Symbol;Acc:1311070]	361602	0,564043379
Atp2b2	ATPase, Ca++ transporting, plasma membrane 2 [Source:RGD Symbol;Acc:2176]	24215	-0,640233763
Ptprz1	protein tyrosine phosphatase, receptor-type, Z polypeptide 1 [Source:RGD Symbol;Acc:3455]	25613	-0,57157327
Isl1	ISL LIM homeobox 1 [Source:RGD Symbol;Acc:61957]	64444	-0,659229193
Ncln	nicalin [Source:RGD Symbol;Acc:1309355]	314648	0,412777931
Col12a1	collagen, type XII, alpha 1 [Source:MGI Symbol;Acc:MGI:88448]	25683	0,516443072
Ltbp4	latent transforming growth factor beta binding protein 4 [Source:RGD Symbol;Acc:1307033]	292734	0,52849614
Krt19	keratin 19, type I [Source:RGD Symbol;Acc:619936]	360626	-0,645142277
Otud1	OTU deubiquitinase 1 [Source:RGD Symbol;Acc:1563344]	498803	0,53303134

Pstpip2	proline-serine-threonine phosphatase-interacting protein 2 [Source:RGD Symbol;Acc:1563090]	307248	-0,504594835
Ski	ski sarcoma viral oncogene homolog (avian) [Source:MGI Symbol;Acc:MGI:98310]		0,427827149
Vasn	vasorin [Source:RGD Symbol;Acc:1598149]	679921	0,401693679
Kcnh1	potassium channel, voltage gated eag related subfamily H, member 1 [Source:RGD Symbol;Acc:68398]	65198	-0,609203098
Cdc25b	cell division cycle 25B [Source:RGD Symbol;Acc:621500]	171103	-0,64212242
Mmp15	matrix metallopeptidase 15 [Source:RGD Symbol;Acc:1308937]	291848	0,476527123
RGD1564664	similar to LOC387763 protein [Source:RGD Symbol;Acc:1564664]	499839	0,670624118
Aim1	absent in melanoma 1 [Source:RGD Symbol;Acc:1308629]		-0,535900335
Clec4b2	C-type lectin domain family 4, member B2 [Source:RGD Symbol;Acc:1359354]	450222	-0,563754051
Slc4a1	solute carrier family 4 (anion exchanger), member 1 [Source:RGD Symbol;Acc:3710]	24779	-0,650046449
Gas2l1	growth arrest-specific 2 like 1 [Source:RGD Symbol;Acc:1311206]	360973	0,423862583
Ifit1lb	interferon-induced protein with tetratricopeptide repeats 1-like B [Source:RGD Symbol;Acc:1304872]	294090	-0,659379736
Gas1	growth arrest-specific 1 [Source:RGD Symbol;Acc:1586820]	683470	0,532880829
Hic1	hypermethylated in cancer 1 [Source:MGI Symbol;Acc:MGI:1338010]	100912068	0,55924126
Kank2	KN motif and ankyrin repeat domains 2 [Source:RGD Symbol;Acc:2320289]	100361376	0,294548799
Ssc5d	scavenger receptor cysteine rich family, 5 domains [Source:RGD Symbol;Acc:1565772]	308341	0,559925078
Bpgm	2,3-bisphosphoglycerate mutase [Source:RGD Symbol;Acc:735018]	296973	-0,60132048
Fdxacb1	ferredoxin-fold anticodon binding domain containing 1 [Source:RGD Symbol;Acc:1562252]	315646	-0,509214967
Ndufs7	NADH dehydrogenase (ubiquinone) Fe-S protein 7 [Source:RGD Symbol;Acc:1310013]	362837	0,364255421
Pik3r2	phosphoinositide-3-kinase, regulatory subunit 2 (beta) [Source:RGD Symbol;Acc:68341]	29741	0,321569195
Wfdc1	WAP four-disulfide core domain 1 [Source:RGD Symbol;Acc:68938]	171112	0,653693676
Slc22a17	solute carrier family 22, member 17 [Source:RGD Symbol;Acc:631405]	305886	0,382131943
Adgra2	adhesion G protein-coupled receptor A2 [Source:RGD Symbol;Acc:2323365]	100363275	0,537146601
Gata4	GATA binding protein 4 [Source:RGD Symbol;Acc:2665]	54254	-0,530072124
Vom2r44	GATA binding protein 4 [Source:RGD Symbol;Acc:2665]	266778	-0,631576948
Mif	macrophage migration inhibitory factor (glycosylation-inhibiting factor) [Source:RGD Symbol;Acc:621163]	81683	0,411506181
AABR07024106.1	LOC103694877; macrophage migration inhibitory factor	103694877	0,411506181
Pxdn	peroxidasin homolog (Drosophila) [Source:MGI Symbol;Acc:MGI:1916925]	554172	0,362152899
Hnrnpa3	heterogeneous nuclear ribonucleoprotein A3 [Source:RGD Symbol;Acc:727807]		-0,429563953
Hic1	hypermethylated in cancer 1 [Source:RGD Symbol;Acc:1307527]		0,549381071
Eno3	enolase 3, beta, muscle [Source:RGD Symbol;Acc:2555]	25438	-0,551379444
Sod3	superoxide dismutase 3, extracellular [Source:RGD Symbol;Acc:3733]	25352	0,458199816
